# Association between the reactivity of local cerebral oxygen saturation after hypo-to-hypercapnic tests and delirium after abdominal surgery in older adults: A prospective study

**DOI:** 10.3389/fpsyt.2022.907870

**Published:** 2022-11-04

**Authors:** Jie Song, Chen Cheng, Kui Sheng, Ling-Ling Jiang, Yun Li, Xiao-Qiong Xia, Xian-Wen Hu

**Affiliations:** ^1^Department of Anaesthesiology, The Second Affiliated Hospital of Anhui Medical University, Hefei, Anhui, China; ^2^Department of Anaesthesiology, The Chaohu Affliated Hospital of Anhui Medical University, Hefei, China

**Keywords:** older adults, abdominal surgery, regional cerebral oxygen saturation (rSO2), delirium, local cerebral blood flow

## Abstract

**Objective:**

This study aimed to investigate the correlation between changes in regional cerebral oxygen saturation (rSO2) and postoperative delirium in older adults undergoing major abdominal surgery.

**Materials and methods:**

This prospective study enrolled older adults scheduled for elective major abdominal surgery at the Second Affiliated Hospital of Anhui Medical University from August 2021 to January 2022. The change in rSO2 from baseline was determined using the hypo-to-hypercapnic test. The main study outcome was the occurrence of postoperative delirium.

**Results:**

A total of 101 participants were included for analysis, of whom 16 (15.8%) developed postoperative delirium. Compared with non-delirium participants, the mean arterial pressure and heart rate were not significantly different in the postoperative delirium group at T0, T1, T2, T3, T4, and T6 (all P_interaction_ > 0.05), but the delirium group had lower pH, lower PaO2, and higher lactate levels at T4, T5, and T6 (all P_interaction_ < 0.05). rSO2 at T0, T1, T2, T3, T4, and T6 was 69.0 (63.2–75.2), 70.7 ± 7.3, 68.2 ± 7.5, 72.1 ± 8.0, 69.9 ± 7.8, 67.4 ± 7.2, and 71.7 ± 8.1, respectively. The postoperative change in rSO2 during the hypercapnia test (TΔrSO2%) was 6.62 (5.31–9.36). Multivariable analysis showed that the Cumulative Illness Rating Scale (odd ratio, OR = 1.89, 95% confidence interval, CI: 1.10–3.25, *P* = 0.021), preoperative albumin levels (OR = 0.67, 95% CI: 0.48–0.94, *P* = 0.022), rSO2 at T4 (OR = 0.61, 95% CI: 0.41–0.89, *P* = 0.010), and postoperative TΔrSO2% (OR = 0.80, 95% CI: 0.66–0.98, *P* = 0.028) were independently associated with postoperative delirium in older adults undergoing elective abdominal surgery.

**Conclusion:**

The rSO2 measured at T4 and postoperative TΔrSO2% were independently associated with postoperative delirium in older adults undergoing elective abdominal surgery.

## Introduction

Older adult patients represent a clinically distinct category of patients with a higher incidence of frailty and comorbidities than young adults, making abdominal surgery challenging in these patients (1–4) due to significantly higher risks of postoperative morbidity and mortality (5). Older adults undergoing abdominal surgery, especially emergency surgery, are prone to postoperative decline that might impact their quality of life (6, 7). As most populations in the world are aging and since life expectancy is increasing, the number of older adults to undergo abdominal surgery is expected to increase (8). Previous literature mainly focused on prolonging life after abdominal surgery, and studies on the impact of postoperative cognitive functions and the ability to perform daily activities by older patients after abdominal surgery are lacking (7, 9).

Delirium is an acute episode of confusion, accompanied by inattention, cognitive impairment, incoherence, and sensory and perceptual abnormalities (10). Postoperative delirium in older adults has been associated with morbidity, mortality, prolonged hospital stays, additional tests, and higher treatment costs (11, 12). The incidence of postoperative delirium in older adults varies from 9 to 87%, depending on the characteristics of the patients and the extent of surgery (13). Thus, early examinations and management are paramount to reducing these associated morbidities (13).

Postoperative delirium is often the result of the interaction of predisposing and precipitating factors. The predisposing factors include ≥65 years of age, cognitive impairment, visual impairment, hearing impairment, history of delirium, functional impairment, history of brain ischemia, comorbidities, depression, alcohol abuse, and hypertension (14, 15). The precipitating factors for postoperative delirium in older adults include recent changes (within hours or days) in behavior, uncontrolled pain, non-judicious use of opioids, polypharmacy, hypoxemia, hypercarbia, metabolic disorders, sepsis, physical restraints, malnutrition, urinary catheterization, abnormalities in fluids and electrolytes, anemia, high blood loss, and transfusion (14, 16, 17). Nevertheless, the interactions among these factors are complex and predicting the occurrence of delirium remains challenging. Scoring systems for predicting delirium were developed and validated but still showed modest predictive ability and often relied on cognitive testing, which can be complicated in the operative setting (18–20).

Intraoperative brain hypoxemia has been suggested to play a major role in the occurrence of postoperative delirium (21–23). Brain hypoxemia can be influenced by changes in regional cerebral oxygen saturation (rSO2), hypotension and hemoglobin levels (24). However, no studies have specifically examined the role of rSO2 in older adults undergoing abdominal surgery. Therefore, this study aimed to investigate the correlation between changes in rSO_2_ and postoperative delirium in older adults undergoing major abdominal surgery as an attempt to predict the occurrence of postoperative delirium by identifying patients who would require early psychological care and management.

## Materials and methods

### Study design and participants

This prospective study enrolled older adults scheduled for elective major abdominal surgery (including radical gastrectomy, radical colorectal cancer resection, partial hepatectomy, and pancreaticoduodenectomy) at the Department of Anesthesiology and Perioperative Medicine of the Second Affiliated Hospital of Anhui Medical University from August 2021 to January 2022. This study was approved by the Ethics Committee for Clinical Trials of the Second Affiliated Hospital of Anhui Medical University (No. YX2021-077 F1). Each participant provided written informed consent. The clinical trial is registered in the China Clinical Trial Registry (CTR2100048072).

Delirium was diagnosed according to the criteria of the Diagnostic and Statistical Manual of Mental Disorders (DSM-5). The inclusion criteria were (1) aged ≥ 60 years old, (2) American Society of Anesthesiologists (ASA) physical status I–III, (3) operative time ≥ 2 h, and (4) written, informed consent was obtained from the patient. The exclusion criteria were (1) unable to receive cognitive and psychological tests for any reason, including sensory impairment, language impairment, or previously diagnosed dementia, (2) cognitive impairment according to the Mini-Mental State Examination (illiteracy ≤ 17 points, primary education ≤ 20 points, secondary education ≤ 22 points, college education ≤ 23 points) (25), (3) participated in another clinical study in the last 3 months, or (4) situations that the investigators considered unsuitable for the study.

### Preoperative examinations

All participants underwent preoperative physical examinations, and the following parameters were recorded: body mass index (BMI), blood pressure, heart rate, American Society of Anesthesiologists score, Numeric Rating Scale for pain assessment, and health habits with special emphasis on smoking, alcohol consumption, and drug use (benzodiazepines, hypnotics, narcotic drugs, and nitrate-containing drugs). The Cumulative Illness Rating Scale (CIRS) was used to assess the comorbidities in each patient (26). The CIRS score was derived from ratings of the impairment of 14 organ systems, with each item scored for severity on a Likert scale (0, no problem; 1, current mild or past significant problem; 2, moderate disability requiring first-line treatment; 3, uncontrollable chronic problems or significant disability; 4, end-organ failure requiring immediate treatment). Within each category, when two diseases were present, only the disease with the highest score was counted. Four score components were obtained for each patient, according to the guidelines of the CIRS: total score, the number of categories endorsed, severity index (total score/number of categories endorsed, and the number of categories at level 3 or 4. The following hematological parameters were measured: hematocrit, red and white blood cell counts, Na+, K+, creatinine, glycemia, albumin, and blood gas analysis [pH, partial pressure of arterial oxygen (PaO2) and carbon dioxide (PaCO2), hemoglobin, and lactate concentration].

### Perioperative management

All patients fasted for 8 h and received no sedative or analgesic medications before anesthesia induction. After arrival in the operation room, vital parameters, including the invasive radial mean arterial pressure (MAP), heart rate, electrocardiogram, pulse oximetry, and end-tidal carbon dioxide partial pressure (PetCO2), were monitored using a Mindray monitor (motor: A7, Mindray Co.). For measuring the rSO2 value, two sensors for near-infrared spectroscopy (INT-100, Hefei ENO Electronics Co.) were placed on the left and right sides of each patient’s forehead. Both their right and left frontal rSO2 values were simultaneously recorded, and the average value of both sides was used for analysis. Patients’ baseline rSO2 data were acquired before anesthesia induction while the patient breathed room air. For bispectral index (BIS) monitoring, a single-use, disposable BIS sensor (Medtronic, Inc., Fridley, MN, USA) was applied to the forehead after the skin was wiped with alcohol swabs.

General anesthesia was induced with sufentanil 0.5 μg/kg and etomidate 0.2 mg/kg, and muscle relaxation was achieved with cisatracurium 0.2 mg/kg. Anesthesia was maintained with sevoflurane (1–2%), propofol (4–8 mg/kg/h), remifentanil (0.1–0.3 μg/kg/min), and cisatracurium (0.1–0.2 mg/kg/h). The BIS values were maintained between 40 and 60. The MAP and heart rate were adjusted using vasoactive agents during the operation based on the appropriate depth of anesthesia and volume therapy. The MAP was maintained within 20% of the baseline value. If the MAP was lower than the target range, phenylephrine 40 μg was administered with further adjustment of 0.1–0.3 μg/kg/min. A urapidil bolus of 0.2–0.5 mg/kg was administered if the MAP was higher than the target range. Similarly, heart rate was adjusted with atropine 0.05 mg if <50 beats/min or esmolol of 20 mg if >100 beats/min. The patient’s body temperature was monitored using a nasal thermometer (Shen Zhen Mecun Healthcare Co.) and maintained between 36.0 and 37.0°C.

Cisatracurium and sevoflurane were terminated about 30 min before the end of the surgery, and sufentanil was administered at 0.2 μg/kg as postoperative analgesia. Propofol and remifentanil were discontinued after the hypercapnia test was performed.

### Hypo-to-hypercapnic test

The hypercapnia test was conducted at the beginning and end of surgery. Approximately 5 min after induction, the tidal volume and ventilation rate were adjusted so that the PaCO2 was maintained at 35–40 mmHg. The rSO2, hemodynamic parameters, and blood gas analysis were recorded as the baseline. Then, the tidal volume and ventilation rate were adjusted so that the PaCO2 was 25–30 mmHg. After maintaining this level for 5 min, the tidal volume was halved, and the ventilation rate was reduced to 2/3, which was maintained until PaCO2 reached 45–50 mmHg and was maintained at this level for about 5 min. After the intervention period, the PaCO2 was maintained at 35–40 mmHg until the end of surgery. The test was repeated at the end of the surgery. The baseline inspiratory gas was 100% oxygen.

This test was based on the reaction of cerebral oxygen saturation from hypocapnia to hypercapnia. Therefore, a patient was considered to have little change when the hypercapnia test did not change the rSO2.

Apart from the baseline data, patients’ blood pressure, rSO2 and blood gas analysis were also recorded at the following endpoints, which were continuously measured and recorded before the induction of anesthesia (T0), 5 min after induction of anesthesia (T1), 5 min after PaCO2 reached 25–30 mmHg (T2), 5 min after PaCO2 reached 45–50 mmHg (T3), at the end of surgery (T4), 5 min after PaCO2 reached 25–30 mmHg again (T5), and 5 min after PaCO2 reached 45–50 mmHg again (T6). The rSO_2_ at T_0_ was considered as baseline, T_1_–T_6_ rSO2 values were collected to compare the effect of carbon dioxide on absolute rSO_2_ value before and after operation.

Postoperative Testing ΔrSO_2_% (change of rSO_2_ during the hypercapnia test) was calculated using the root mean of successive squared differences (RMSSD) (27) of averaged 1 min rSO2% values through the testing time. The formula was implant into the near-infrared spectroscopy:


$$RMSSD=\sqrt{\frac{\sum_{i=2}^{n}{\left(Xi-Xi-1\right)}^{2}}{n}}$$

Where Xi is the rSO2 at time i.

### Outcomes and follow-up

The primary outcome of the study was the incidence of delirium within 3 days after the surgery, which was assessed by trained staff at 12, 24, 48, and 72 h postoperatively. The Nursing Delirium Screening Scale (Nu-DESC) was used for preliminary screening postoperative delirium (28). The Nu-DESC is scored on five symptoms (disorientation, inappropriate behavior, inappropriate communication, illusions/hallucinations, and psychomotor retardation). Each item is scored 0–2 points according to the severity, of which 10 represents the maximum total points. If the patient with a total score ≧ 2, then delirium was diagnosed and graded by a professional physician according to the diagnostic criteria in the Diagnostic and Statistical Manual of Mental Disorders, Edition 5 (28).

### Sample size

The sample size was calculated according to the primary outcome, i.e., the incidence of postoperative delirium in patients with reduced rSO2 change rate after major abdominal surgery detected by the hypercapnia test. According to previous studies, comparing PetCO2 30–35 mmHg, the rSO2 at PetCO2 40–45 mmHg was increased by 5–6%, and it was assumed that a decrease of <5% indicated little change (29). In a previous study, the incidence of postoperative delirium (POD) was 26% at an rSO2 change of <5%. Using a unilateral F test (significance level of 0.05) for 95 subjects, the difference detection ability of the α coefficient under the original hypothesis 0.50 and alternative hypothesis 0.26 reached 80%. After estimating a dropout rate of 10%, an estimated sample size of 104 patients was needed.

### Statistical analysis

The Statistical Package for Social Sciences software (SPSS, v22.0; IBM, Armonk, NY, USA) was used for statistical analyses. The Kolmogorov–Smirnov test was used to test the normality of continuous data at *n* > 50, and the Shapiro–Wilk test at *n* < 50. The continuous data complying with a normal distribution were expressed as mean ± standard deviation, and those complying with a non-normal distribution were expressed as a median (25th percentile, 75th percentile). The categorical data were expressed as *n* (%). Repeated and continuous data were analyzed using the generalized estimating equation method. Logistic regression was used for univariable/multivariable regression with the outcome of delirium. Pearson correlation analysis was used for the collinearity of continuous variables. α = 0.05, *P*-values < 0.05 were considered statistically significant. A *post hoc* power calculation was applied to determine the resultant power of the study. The power was calculated by using G*Power 3.1 software.

## Results

### Characteristics of the participants

Of the 139 participants initially assessed, 35 were excluded, and three were lost to follow-up. Therefore, 101 participants were included in the final analysis. The baseline characteristics of the participants are shown in Table 1. The participants were 66.5–78.0 years of age, 68.3% of the participants were male, and the median CIRS score of the whole cohort was 8 (range, 7–11). Significant hypotension was observed in 43.6% of the cases, and median blood loss was 600 ml (range, 500–600).

**TABLE 1:** Baseline characteristics of the 101 included participants.

Characteristics				Value
Sex, *n* (%)	
Female				32 (31.7%)
Male				69 (68.3%)
Age (years) (mean ± range)				73.0 (66.5, 78.0)
History of alcohol abuse, *n* (%)				6 (5.9%)
ASA ≥ III, *n* (%)				12 (11.9%)
CIRS, median (Q25–Q75)				8.0 (7.0, 11.0)
BMI (kg/m^2^), mean ± SD				22.2 ± 2.8
Intraoperative moderate or severe hypotension, *n* (%)				44 (43.6%)
Vasopressor use, *n* (%)				63 (62.4%)
Blood loss (ml), median (Q25–Q75)				600 (400, 600)
Perioperative blood transfusion, median (Q25–Q75)				0 (0, 0)
Urine output (ml), median (Q25–Q75)				600 (500, 1000)
Duration of anesthesia (min), mean ± SD				353.4 ± 94.5
Surgical procedures, *n* (%)				
Gastric or intestinal surgery				53 (52.5%)
Pancreaticoduodenectomy				22 (21.8%)
Partial hepatectomy				26 (25.7%)
Preoperative blood gas analysis				
pH, mean ± SD				7.42 ± 0.04
PaO2/FiO2, median (Q25–Q75)				400.0 (373.5, 465.0)
PaCO2, median (Q25–Q75)				40.0 (37.0, 45.0)
BE, median (Q25–Q75)				2.2 (1.3, 4.0)
Lactate, median (Q25–Q75)				0.90 (0.60, 1.10)
Preoperative Na+ (mmol/L), median (Q25–Q75)				138.0 (137.0, 140.0)
Preoperative K+ (mmol/L), median (Q25–Q75)				4.1 (3.8, 4.2)
Preoperative creatinine (mmol/L), median (Q25–Q75)				59.0 (50.0, 69.0)
Preoperative Hb (g/L), mean ± SD				111.3 ± 18.3
Preoperative Hematocrit, median (Q25–Q75)				36.0 (31.0, 38.0)
Preoperative white blood cell count (× 10^9^/L), median (Q25–Q75)				6.01 (4.85, 7.50)
Preoperative albumin (g/dl), mean ± SD				38.7 ± 5.5
Length of hospital stay (d), median (Q25–Q75)				15.0 (13.0, 18.0)
Perioperative NRS, median (Q25–Q75)				0.0 (0.0, 0.0)
Preoperative NRS, median (Q25–Q75)				
6 h				1.0 (1.0, 3.0)
1 day				1.0 (0.0, 2.0)
2 days				0.0 (0.0, 1.0)
3 days				0.0 (0.0, 1.0)

**Characteristics**	**Total cases**	**Delirium cases**	**Non-delirium cases**	***P*-value**

Sex, *n* (%)				
Female	32 (31.7%)	7 (43.75%)	25 (29.41%)	
Male	69 (68.3%)	9 (56.25%)	60 (70.59%)	
Age (years), median (Q25–Q75)	73.0 (66.5, 78.0)	75 (72, 80.5)	72 (65, 77)	0.034
History of alcohol abuse, *n* (%)	6 (5.9%)	2 (12.5%)	4 (4.71%)	0.226
ASA ≥ III, *n* (%)	46 (45.5%)	5 (31.25%)	41 (48.24%)	0.396
CIRS, median (Q25–Q75)	8.0 (7.0, 11.0)	11.5 (7.5,14)	7 (7, 10)	0.001
BMI (kg/m^2^), mean ± SD	22.2 ± 2.8	22.79 ± 3.26	22.11 ± 2.67	0.371
Intraoperative moderate or severe hypotension, *n* (%)	44 (43.6%)	9 (56.25%)	35 (41.18%)	0.265
Vasopressor use, *n* (%)	63 (62.4%)	14 (87.5%)	49 (57.65%)	0.024
Blood loss (ml), median (Q25–Q75)	600 (400, 600)	600 (450,800)	600 (400, 600)	0.337
Perioperative blood transfusion, median (Q25–Q75)	0 (0, 0)	0 (0,300)	0 (0,0)	0.107
Urine output (ml), median (Q25–Q75)	600 (500, 1000)	750 (500,1000)	600 (500, 900)	0.570
Duration of anesthesia (min), mean ± SD	353.4 ± 94.5	341.88 ± 70.65	355.55 ± 98.51	0.598
Surgical procedures, *n* (%)				
Gastric or intestinal surgery	53 (52.5%)	8 (50%)	45 (52.94%)	0.849
Pancreaticoduodenectomy	22 (21.8%)	3 (18.75%)	19 (22.35%)	
Partial hepatectomy	26 (25.7%)	5 (31.25%)	21 (24.71%)	
Preoperative blood gas analysis				
pH, mean ± SD	7.42 ± 0.04	7.41 ± 0.04	7.42 ± 0.04	0.654
PaO2/FiO2, median (Q25–Q75)	400.0 (373.5, 465.0)	382.5 (344.5, 464)	409 (376, 465)	0.276
PaCO2, median (Q25–Q75)	40.0 (37.0, 45.0)	44.5 (38.5, 48)	39 (36, 45)	0.121
BE, median (Q25–Q75)	2.2 (1.3, 4.0)	1.65 (0.8, 3.3)	2.4 (1.5, 4)	0.171
Lactate, median (Q25–Q75)	0.90 (0.60, 1.10)	0.9 (0.65, 1.15)	0.9 (0.6, 1.1)	0.630
Preoperative Na+ (mmol/L), median (Q25–Q75)	138.0 (137.0, 140.0)	140 (137, 142.5)	138 (137, 140)	0.086
Preoperative K+ (mmol/L), median (Q25–Q75)	4.1 (3.8, 4.2)	3.85 (3.8, 4.15)	4.1 (3.8, 4.3)	0.088
Preoperative creatinine (mmol/L), median (Q25–Q75)	59.0 (50.0, 69.0)	63.5 (55.5, 71)	59 (50, 68)	0.173
Preoperative Hb (g/L), mean ± SD	111.3 ± 18.3	107 (99, 121)	112 (100, 121)	0.493
Preoperative Hematocrit, median (Q25–Q75)	36.0 (31.0, 38.0)	35 (30, 37.5)	36 (31, 39)	0.351
Preoperative white blood cell count (× 10^9^/L), median (Q25–Q75)	6.01 (4.85, 7.50)	5.89 (4.45, 7.6)	6.09 (5.02, 7.33)	0.586
Preoperative albumin (g/dl), mean ± SD	38.7 ± 5.5	35.99 ± 7.14	39.21 ± 5.02	0.031
Length of hospital stay (d), median (Q25–Q75)	15.0 (13.0, 18.0)	17 (14, 21)	15 (13, 18)	0.087
Perioperative NRS ≥ 3, *n* (%)				
Preoperative	13 (12.87%)	2 (12.5%)	11 (12.94%)	0.961
6 h	16 (15.84%)	3 (18.75%)	13 (15.29%)	0.759
1 day	30 (29.7%)	4 (17.65%)	26 (36.48%)	0.629
2 days	17 (16.83%)	2 (12.5%)	15 (17.65%)	0.759
3 days	9 (10.59%)	1 (6.25%)	8 (9.41%)	0.849

ASA, American Society of Anesthesiologists; CIRS, cumulative illness rating scale; BMI, body mass index; NRS, numeric rating scale; PaO2, partial arterial oxygen pressure; PaCO2, partial arterial carbon dioxide pressure; rSO2, local cerebral oxygen saturation; Q, percentile.

### Regional cerebral oxygen saturation

Table 2 presents the rSO2 data at various time points. The rSO2 was 69.3 ± 6.8 at T0, 70.7 ± 7.3 at T1, 68.2 ± 7.5 at T2, 72.1 ± 8.0 at T3, 69.9 ± 7.8 at T4, 67.4 ± 7.2 at T5, and 71.7 ± 8.1 at T6. Hence, the postoperative TΔrSO2% was 6.62 (Inter-Quartile Range, IQR: 5.31–9.36).

**TABLE 2:** Local cerebral oxygen saturation (rSO2) values at different time points.

Characteristics	Total cases	Delirium cases	Non-delirium cases	Value
rSO2 %				
T0, mean ± SD	69.3 ± 6.8	69.9 ± 7.3	69.3 ± 6.8	0.753
T1, mean ± SD	70.7 ± 7.3	65.4 ± 7.3	71.7 ± 6.9	0.001
T2, mean ± SD	68.2 ± 7.5	67.6 ± 8.6	68.4 ± 7.3	0.731
T3, mean ± SD	72.1 ± 8.0	62.8 ± 6.0	73.9 ± 7.1	0.000
T4, mean ± SD	69.9 ± 7.8	63.2 ± 6.0	70.9 ± 7.1	0.000
T5, mean ± SD	67.4 ± 7.2	64.5 ± 8.5	67.9 ± 6.8	0.078
T6, mean ± SD	71.7 ± 8.1	63.7 ± 6.4	73.2 ± 7.5	0.000
Postoperative TΔrSO2%, median (Q25–Q75)	6.62 (5.31, 9.36)	0.02 (-0.11, 0.11)	0.07 (0.06, 0.09)	0.041

### Postoperative delirium

Sixteen (15.8%) participants were screened positive by nurses and were finally diagnosed with postoperative delirium. Compared with the non-delirium participants, the MAP and HR were not significantly different in the postoperative delirium group at all time points (all P_interaction_ > 0.05). However, the delirium group had significantly lower pH at T4, T5 and T6 (all P_interaction_ < 0.05), significantly lower PaO2 at T4, T5, and T6 (all P_interaction_ < 0.05), and significantly higher lactate levels at T4, T5, and T6 (all P_interaction_ < 0.05) (Table 3).

**TABLE 3:** Comparison of the characteristics between the postoperative delirium (POD) and non-POD groups at multiple time points.

Variable	Time	POD (*n* = 16)	NPOD (*n* = 85)	P_Time_	P_Group_	P_Interaction_
MAP	T0	96.19 ± 12.69	100.75 ± 15.50	Ref	0.192	Ref
	T1	82.94 ± 10.19	83.18 ± 8.29	< 0.001		0.374
	T2	80.94 ± 9.15	83.22 ± 8.52	< 0.001		0.627
	T3	79.50 ± 8.73	82.65 ± 9.84	< 0.001		0.739
	T4	79.25 ± 11.20	82.28 ± 8.48	< 0.001		0.741
	T5	77.63 ± 7.91	81.46 ± 9.78	< 0.001		0.859
	T6	79.19 ± 7.89	81.95 ± 9.21	< 0.001		0.671
HR	T0	72.56 ± 10.92	72.07 ± 9.67	Ref	0.863	
	T1	67.81 ± 9.03	69.18 ± 12.72	0.104		0.474
	T2	68.13 ± 7.80	69.34 ± 9.02	0.036		0.417
	T3	66.69 ± 8.99	69.32 ± 10.65	0.026		0.187
	T4	65.75 ± 11.71	67.18 ± 10.74	0.001		0.489
	T5	63.88 ± 8.20	67.24 ± 11.41	0.002		0.262
	T6	63.00 ± 10.35	66.68 ± 9.52	< 0.001		0.220
pH	T0	7.37 ± 0.06	7.36 ± 0.07	Ref	0.776	
	T1	7.36 ± 0.06	7.36 ± 0.05	0.603		0.994
	T2	7.36 ± 0.05	7.38 ± 0.06	0.095		0.336
	T3	7.36 ± 0.04	7.36 ± 0.05	0.878		0.485
	T4	7.29 ± 0.04	7.35 ± 0.05	0.092		0.002
	T5	7.29 ± 0.07	7.35 ± 0.05	0.061		0.039
	T6	7.27 ± 0.06	7.35 ± 0.06	0.052		0.002
PaO2	T0	419.06 ± 63.24	423.73 ± 68.83	Ref	0.784	
	T1	428.50 ± 68.42	423.05 ± 68.74	0.810		0.498
	T2	410.13 ± 75.76	417.53 ± 77.01	0.124		0.878
	T3	402.56 ± 76.95	415.28 ± 70.83	0.072		0.658
	T4	374.56 ± 57.20	416.56 ± 71.10	0.347		0.041
	T5	364.50 ± 52.17	416.72 ± 67.26	0.179		0.001
	T6	361.69 ± 55.83	414.85 ± 61.30	0.068		0.001
LAC	T0	1.65 ± 1.30	1.71 ± 0.98	Ref	0.844	
	T1	1.84 ± 0.97	1.73 ± 1.26	0.197		0.738
	T2	1.78 ± 0.97	1.68 ± 1.27	0.580		0.822
	T3	1.81 ± 0.88	1.68 ± 1.31	0.617		0.698
	T4	1.84 ± 1.32	2.84 ± 1.58	0.136		0.023
	T5	1.86 ± 1.33	2.93 ± 1.66	0.100		0.028
	T6	1.89 ± 1.30	2.93 ± 1.60	0.068		0.029

**Variable**	**Time**	**POD**		***t***	***p***
		**NPOD**	**POD**		

MAP	T0	100.7529 ± 15.4995	96.1875 ± 12.6871	1.109	0.270
	T1	83.1765 ± 8.2882***	82.9375 ± 10.188*	0.102	0.919
	T2	83.2235 ± 8.5194***	80.9375 ± 9.154**	0.973	0.333
	T3	82.6471 ± 9.84***	79.5 ± 8.7254***	1.193	0.236
	T4	82.2824 ± 8.4819***	79.25 ± 11.1982**	1.244	0.217
	T5	81.4588 ± 9.784***	77.625 ± 7.9067***	1.477	0.143
	T6	81.9529 ± 9.2104***	79.1875 ± 7.8928***	1.125	0.263
HR	T0	72.0706 ± 9.673	72.5625 ± 10.9238	–0.183	0.855
	T1	69.1765 ± 12.715	67.8125 ± 9.0349*	0.409	0.683
	T2	69.3412 ± 9.0232*	68.125 ± 7.8049*	0.504	0.615
	T3	69.3176 ± 10.6528*	66.6875 ± 8.9942*	0.926	0.357
	T4	67.1765 ± 10.7362**	65.75 ± 11.7104*	0.481	0.632
	T5	67.2353 ± 11.4087**	63.875 ± 8.1965*	1.404	0.172
	T6	66.6824 ± 9.5197***	63 ± 10.3473**	1.400	0.165
PH	T0	7.3632 ± 0.0714	7.3681 ± 0.0645	–0.258	0.797
	T1	7.3595 ± 0.0538	7.3644 ± 0.0628	–0.322	0.748
	T2	7.3759 ± 0.0621	7.3594 ± 0.0528	0.996	0.322
	T3	7.3645 ± 0.0469	7.355 ± 0.0431	0.750	0.455
	T4	7.3493 ± 0.0486	7.2913 ± 0.0363**	4.536	0.000
	T5	7.3479 ± 0.0504	7.2931 ± 0.0737*	3.682	0.000
	T6	7.3462 ± 0.0554	7.2719 ± 0.0639**	4.810	0.000
PaO2	T0	423.7294 ± 68.8287	419.0625 ± 63.2439	0.252	0.802
	T1	423.0471 ± 68.7353	428.5 ± 68.4222	–0.291	0.771
	T2	417.5294 ± 77.0104	410.125 ± 75.76	0.354	0.724
	T3	415.2824 ± 70.8311	402.5625 ± 76.9484	0.650	0.517
	T4	416.5647 ± 71.1035	374.5625 ± 57.2002*	2.228	0.028
	T5	416.7176 ± 67.2587	364.5 ± 52.1715**	2.939	0.004
	T6	414.8471 ± 61.298	361.6875 ± 55.8304**	3.224	0.002
LAC	T0	1.6518 ± 1.2957	1.7063 ± 0.9849	–0.159	0.874
	T1	1.7247 ± 1.2607	1.8375 ± 0.9743	–0.339	0.735
	T2	1.6824 ± 1.2711	1.775 ± 0.9733	–0.276	0.783
	T3	1.68 ± 1.3053	1.8063 ± 0.8835	–0.370	0.712
	T4	1.8353 ± 1.321	2.8875 ± 1.5764*	–2.833	0.006
	T5	1.8576 ± 1.3335	2.925 ± 1.6631*	–2.821	0.006
	T6	1.8859 ± 1.3026	2.9313 ± 1.5974*	–2.839	0.005
MAP	T0	100.7529 ± 15.4995	96.1875 ± 12.6871	1.109	0.270
	T1	83.1765 ± 8.2882***	82.9375 ± 10.188*	0.102	0.919
	T2	83.2235 ± 8.5194***	80.9375 ± 9.154**	0.973	0.333
	T3	82.6471 ± 9.84***	79.5 ± 8.7254***	1.193	0.236
	T4	82.2824 ± 8.4819***	79.25 ± 11.1982**	1.244	0.217
	T5	81.4588 ± 9.784***	77.625 ± 7.9067***	1.477	0.143
	T6	81.9529 ± 9.2104***	79.1875 ± 7.8928***	1.125	0.263

T0: time before the induction of anesthesia; T1: 5 min after induction of anesthesia; T2: 5 min after PaCO2 reached 25–30 mmHg; T3: 5 min after PaCO2 reached 45–50 mmHg; T4: at the end of surgery; T5: 5 min after PaCO2 reached 25–30 mmHg again; T6: 5 min after PaCO2 reached 45–50 mmHg again. POD, postoperative delirium; NPOD, no postoperative delirium; MAP, mean arterial pressure; HR, heart rate; PaO2, partial arterial oxygen pressure; LAC, lactate. Compare to T0 *p < 0.1, **P < 0.05, ***P < 0.001.

### Factors associated with postoperative delirium

The univariable and multivariable analyses of delirium are shown in Table 4. Univariate analysis showed that age (*P* = 0.028), CIRS (*P* = 0.002), vasopressor use (*P* = 0.037), preoperative albumin levels (*P* = 0.038), length of hospital stay (*P* = 0.018), rSO2 at T1 (*P* = 0.003), rSO2 at T4 (*P* < 0.001) and postoperative TΔrSO2% (*P* = 0.002) were significantly associated with postoperative delirium. However, on multivariate analysis, CIRS (OR = 1.89, 95% CI: 1.10–3.25, *P* = 0.021), preoperative albumin levels (OR = 0.67, 95% CI: 0.48–0.94, *P* = 0.022), rSO2 at T4 (OR = 0.61, 95% CI: 0.41–0.89, *P* = 0.010) and TΔrSO2% (OR = 0.80, 95% CI: 0.66–0.98, *P* = 0.028) were found to be independently associated with postoperative delirium in older adults undergoing elective abdominal surgery.

**TABLE 4:** Univariate and multivariable analysis of factors associated with delirium.

Variables	Univariable analysis		Multivariable analysis	
	OR (95% CI)	*P*	OR (95% CI)	*P*
Age	1.110 (1.011, 1.217)	0.028	1.199 (0.911, 1.577)	0.195
Sex (female vs. male)	1.867 (0.626, 5.565)	0.263		
History of alcohol abuse	2.893 (0.483, 17.321)	0.245		
ASA ≥ III	1.949 (0.465, 8.167)	0.361		
CIRS	1.312 (1.107, 1.555)	0.002	1.894 (1.102, 3.254)	0.021
BMI	1.094 (0.900, 1.331)	0.368		
Intraoperative moderate or severe hypotension	1.837 (0.625, 5.398)	0.269		
Vasopressor use (yes vs. no)	5.143 (1.099, 24.057)	0.037	5.224 (0.365, 74.732)	0.223
Blood loss	1.002 (0.999, 1.004)	0.165		
Perioperative blood transfusion	1.002 (1.000, 1.005)	0.086		
Urine output	1.001 (0.999, 1.002)	0.422		
Duration of anesthesia	0.998 (0.992, 1.004)	0.594		
Surgical procedures				
Gastric or Intestinal surgery	0.747 (0.218, 2.559)	0.642		
Pancreaticoduodenectomy	0.663 (0.139, 3.156)	0.606		
Partial hepatectomy	ref			
Preoperative blood gas analysis				
pH	0.044 (0.000, 31,960.383)	0.650		
PaO2/FiO2	0.996 (0.988, 1.005)	0.389		
PaCO2	1.050 (0.976, 1.130)	0.187		
BE	0.879 (0.695, 1.112)	0.282		
Lactate	2.032 (0.649, 6.359)	0.223		
Preoperative Na+	1.123 (0.984, 1.283)	0.086		
Preoperative K+	0.292 (0.069, 1.230)	0.094		
Preoperative creatinine level	1.013 (0.978, 1.050)	0.461		
Preoperative Hb	0.991 (0.962, 1.021)	0.561		
Preoperative hematocrit	0.947 (0.862, 1.042)	0.264		
Preoperative white blood cell count	0.892 (0.679, 1.171)	0.410		
Preoperative albumin	0.902 (0.818, 0.994)	0.038	0.669 (0.475, 0.942)	0.022
Length of hospital stay	1.210 (1.034, 1.417)	0.018	1.068 (0.686, 1.664)	0.770
Perioperative NRS ≥ 3				
Preoperative	0.961 (0.192, 4.814)	0.961		
6 h	0.907 (0.562, 1.465)	0.691		
1 day	0.796 (0.499, 1.271)	0.339		
2 days	0.796 (0.431, 1.469)	0.465		
3 days	0.330 (0.073, 1.489)	0.149		
rSO2 %				
T0	1.013 (0.936, 1.096)	0.750		
T1	0.864 (0.784, 0.952)	0.003		
T4	0.800 (0.719, 0.890)	<0.001	0.605 (0.413, 0.887)	0.010
Postoperative TΔrSO2%	0.848 (0.763, 0.942)	0.002	0.803 (0.659, 0.977)	0.028

A large difference in the number of people between the two groups, resulting in a large OR value. ASA, American Society of Anesthesiologists; CIRS, cumulative illness rating scale; BMI, body mass index; NRS, numeric rating scale; PaO2, partial arterial oxygen pressure; PaCO2, partial arterial carbon dioxide pressure; rSO2, local cerebral oxygen saturation.

## Discussion

This study investigated factors, particularly changes in rSO_2_, to help predicting the occurrence of postoperative delirium in older adults (≥60 years old) from a cohort of 101 patients undergoing major abdominal surgery. Overall, our results suggested that rSO2 measured after the hypercapnia test at the end of surgery and postoperative TΔrSO2% were independently associated with postoperative delirium in older adults undergoing elective abdominal surgery. Considering that the risk of death increases by 11% for every 48 h of active delirium (30), we believe that these indicators could be used as a practical approach for predicting the occurrence of postoperative delirium and identifying patients who could require early psychological care and management.

A decrease in blood pressure, oxygen saturation, and/or hemoglobin was previously reported to influence brain rSO2 (24), a marker of brain oxygenation that comprehensively includes multiple parameters simultaneously. In this regard, Pedrini et al. (31) showed that rSO2 could detect brain hypoperfusion during carotid revascularization with 100% sensitivity and 91% specificity, but they did not examine the occurrence of postoperative delirium. Two other studies showed that rSO2 could predict delirium after cerebral endovascular (32) and on-pump cardiac surgeries (33). However, these two types of surgeries have already been associated with high rates of postoperative delirium (34, 35). Eertmans et al. (36) showed that rSO2 was associated with delirium in older adults after cardiac surgery. However, related studies on older patients undergoing abdominal surgeries have been missing. Thus, we investigated the association between changes in rSO2 after hypo-to-hypercapnic tests and delirium in older adults undergoing elective abdominal surgery including radical gastrectomy, radical colorectal cancer resection, partial hepatectomy, and pancreaticoduodenectomy. The results showed that the incidence of postoperative delirium was 16%, and rSO2 was independently associated with postoperative delirium, which was physiologically reliable and consistent with previous studies (37). There are several methods via which for assessing cerebral blood flow could be clinical measured during surgery. In this present study, we used rSO2 due to its easy availability, cheap cost, easier to determine and high accuracy. Comparatively, Ortega-Loubon et al. (38) performed a meta-analysis to investigate near-infrared spectroscopy monitoring in cardiac and non-cardiac surgery and found that rSO2-based algorithms could help prevent delirium after cardiac surgery but not after non-cardiac surgeries, indicating the need for additional studies on rSO2 in non-cardiac surgery settings to validate these results.

Intraoperative cerebral oxygen desaturation has been proposed as a possible mechanism of postoperative cognitive dysfunction (21–23). In this study, we also verified the hypothesis that a decrease in rSO2 change rate during a hypercapnic test is a risk factor for postoperative delirium. In this study, TΔrSO2% was 6.62%, and our results showed that the T4–T1 difference in rSO2 was not associated with delirium, but the rSO2 at the end of surgery (i.e., TΔrSO2%) was associated with postoperative delirium. It is supported by previous studies which showed that brain hypoxemia during surgery was associated with postoperative delirium (21–23). Wang et al. (32) used the rSO2 desaturation score, calculated based on baseline rSO2 and changes in rSO2 over time (39–41), and showed that the rSO2 desaturation score was associated with postoperative delirium. Similar results were observed in patients undergoing cardiac surgery (42). Casati et al. (41) reported that rSO2 desaturation was associated with early postoperative cognitive decline. Still, the rSO2 desaturation score requires continuous rSO2 measurements and calculations, which might be impractical in the actual operating room setting. On the other hand, Lin et al. (43) suggested that an intraoperative decrease in the rSO2 was not associated with postoperative delirium in digestive cancer patients undergoing laparoscopy. In our study, the rSO2 stability in the POD group was poor after induction, which may be related to the use of vasoactive agents after blood pressure fluctuation. At the same time, increased intracranial pressure and decreased cerebral blood flow due to carbon dioxide cannot be ruled out at T3. Statistical analysis showed that rSO2 were significantly lower in POD group than in the normal neurocognitive function group at the end of the surgery. The difference was less likely to be elevated by carbon dioxide, which takes more consideration of other influencing factors, such as pH, PaO2, and Lac, etc. Thus, the present study suggested that the difference in rSO2 values between two predetermined time points might achieve a similar predictive value. Discrepancies among studies might depend upon the type of surgery, the patient population, and the time points selected for rSO2 measurement. Still, the extent of change in the rSO2 after surgery remains to be validated in future studies. The predictive value will have to be determined using receiver operating characteristics curves to determine the optimal cutoff value, sensitivity, and specificity in a large group of patients.

In previous studies, the relationship between cerebral blood flow (CBF) and PaCO_2_ changes is known as cerebral vasomotor reactivity (CVMR), which is often used to assess cerebrovascular function in research and clinical Settings (44). The basal value of rSO_2_ varies greatly between individuals, as does the reactivity to carbon dioxide. The history of diabetes mellitus (DM) and hypertension (HTN) in elderly patients is an important factor influencing the different reactivity of PaCO_2_ to rSO_2_. Such diseases can cause changes in cerebrovascular structure and function, such as increased vessel wall lumen ratio and decreased vasoconstriction and vasodilation ability of cerebral arterioles (45, 46). Therefore, cerebrovascular CO_2_ reactivity is more impaired in those patients than in normal patients. McCulloch found that hypercapnia impaired brain self-regulation during general anesthesia, and this difference resulted in a differential rSO_2_ response to CO2 (47). CVMR in those frail patients is susceptible to many factors, such as age, surgical site, anesthesia mode, and comorbid diseases, that would explain the result for TΔrSO_2_%.

Of note, the rSO2 was measured during a hypercapnia test, and whether permissive hypercapnia has brain protection effects remains controversial. Permissive hypercapnia might have a protective effect in patients with a high rate of response change, but in patients who already have an impaired vascular response, permissive hypercapnia and resulting acidemia might limit brain protection. Therefore, additional studies are needed to examine the effect of the hypercapnia test used to assess the rSO2.

The underlying etiology of delirium still remains unknown. However, it is believed that a large amount of blood loss during major surgery, such as gastrointestinal surgery, may cause disbalances (i.e., homeostasis, hemodynamics, etc.) in important organs (48). Cerebral oxygen metabolism can reflect the relationship between oxygen supply and consumption in the brain, and the state of cerebral circulation, which are also considered an important basis for early detection of cerebral ischemia and hypoxia. Previous studies have shown that postoperative cognitive dysfunction in elderly patients with non-cardiac surgery might be related to abnormal cerebral oxygen metabolism during surgery (49, 50). Neuropathological studies indicated that diffuse neuronal ischemia might primarily affect brain areas susceptible to hypoxic-ischemic injuries, such as watershed areas in the frontal cortex (51). Therefore, poor cerebral perfusion may contribute to delirium. In this study, we identified that CIRS and preoperative albumin levels were also associated with postoperative delirium. The CIRS is a measure of the comorbidity load in patients (26), while albumin levels represent the nutritional status of patients (52). Hypoalbuminemia may result from blood loss from surgeries, trauma, insufficient food intake, stress reaction, and other reasons (53). The mechanism of hypoalbuminemia leads to delirium is unclear; however, it may be assumed that albumin, apart from transporting various trace elements and drugs, may also have functions associated with antioxidation, scavenging free radicals and protecting the microcirculation, playing a vital role in the body’s metabolism (54). Clinically, strategies targeting low albumin levels in elderly patients have been implanted and have shown promising result in reducing the risk of POD (55).

Clinically, the prediction of delirium in older patients is very important because apart from reducing financial burdens to the patients, it helps improve the treatment outcomes of the patients and reduce psychological burdens on the patients, their relatives and carers as well. In addition, it is crucial that delirium care plans also involve non-pharmacological measures. Studies have shown that using non-pharmacological treatment with polygenic strategies can reduce the incidence of delirium in patients (56, 57), thereby reducing the cost to the health care establishment. Overall, by predicting the incidence of delirium preoperatively, identifying markers that could timely detect the occurrence of delirium and strengthening early perioperative nursing management and early delirium treatment, this would ultimately reduce medical costs and allow rapid recovery of the patients.

This study had limitations. The study was performed at a single center. Additional randomized clinical trials with large sample sizes are needed to provide a higher level of evidence. Second, although the gold standard for assessing cerebral blood flow is via Doppler ultrasound; however, considering it is more expensive, time-consuming and invasive than rSO2, this study used rSO2 because it was easier to perform and had high accuracy. Third, many factors, including age, hemodynamics, body temperature, inhaled oxygen concentration, pH, etc., may affect rSO2. This study recorded and compared age, hemodynamics, and pH and controlled the body temperature and inhaled oxygen concentration to 100%. Additional studies should examine the impact of different operative conditions on rSO2 and their association with postoperative delirium occurrence.

In conclusion, rSO2 measured after a hypo-to-hypercapnia test at the end of surgery and postoperative TΔrSO2% were independently associated with postoperative delirium in older adults undergoing elective abdominal surgery. The CIRS and preoperative albumin levels were also independently associated with postoperative delirium. Future studies should examine whether the hypercapnic test could have a protective effect in some patients.

## Data availability statement

The original contributions presented in the study are included in the article/supplementary material, further inquiries can be directed to the corresponding authors.

## Ethics statement

The studies involving human participants were reviewed and approved by the Ethics Committee of the Second Affiliated Hospital of Anhui Medical University (No. YX2021-077 F1). The patients/participants provided their written informed consent to participate in this study.

## Author contributions

JS and X-WH: conception and design of the research and writing of the manuscript. CC and KS: acquisition of data. L-LJ: analysis and interpretation of the data and statistical analysis. X-WH: obtaining financing. YL, X-WH, and X-QX: critical revision of the manuscript for intellectual content. All authors read and approved the final draft.

## Abbreviations

rSO2: local cerebral oxygen saturation
ASA: American Society of Anesthesiologists
CIRS: cumulative illness rating scale
BMI: body mass index
NRS for pain assessment: numeric rating scale for pain assessment
PaO2: partial arterial oxygen pressure
PaCO2: partial arterial carbon dioxide pressure
EtCO2: end-tidal carbon dioxide partial pressure
POD: postoperative delirium
NPOD: no postoperative delirium
MAP: mean arterial pressure
HR: heart rate
LAC: lactate
pH: potential of hydrogen
BE: base excess
SD: standard deviation
OR: odd ratio
CI: confidence interval
GEE: the generalized estimation equation method
Nu-DESC: the nursing delirium screening scale
DSM: the diagnostic and statistical manual of mental disorders
MMSE: the mini-mental state examination
BIS: bispectral index.
